# Supplementary figures and images for: Effects of fishing restrictions on the recovery of the endangered Saimaa ringed seal (Pusa hispida saimensis) population
Source: PLoS One. 2024 Dec 5;19(12):e0311255. doi: 10.1371/journal.pone.0311255 (PMC11620628; doi:10.1371/journal.pone.0311255)

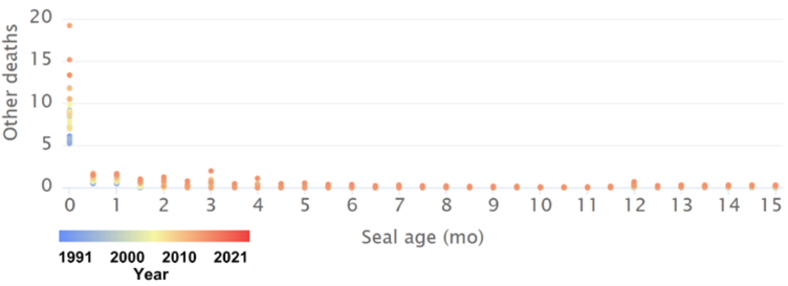

Supplement: S1 Fig — (TIF) [file pone.0311255.s001.tif]
