# Supplementary material for: Effects of fishing restrictions on the recovery of the endangered Saimaa ringed seal (Pusa hispida saimensis) population
Source: PLoS One. 2024 Dec 5;19(12):e0311255. doi: 10.1371/journal.pone.0311255 (PMC11620628; doi:10.1371/journal.pone.0311255)
Supplement: S1 Table — (DOCX) [file pone.0311255.s002.docx]

**S2 Table.** Estimates of Fig 3.

| **Year** | **Gillnet ban until 1st August = Seal age 5 months** | **Gillnet ban until 1st October = Seal age 7 months** | **Year-round gillnet ban = Seal age 15 months** |
| --- | --- | --- | --- |
| **1991** | 0.20 | 0.39 | 0.98 |
| **1992** | 0.21 | 0.43 | 0.92 |
| **1993** | 0.22 | 0.48 | 1.36 |
| **1994** | 0.25 | 0.44 | 1.46 |
| **1995** | 0.24 | 0.34 | 1.25 |
| **1996** | 0.26 | 0.50 | 1.30 |
| **1997** | 0.37 | 0.79 | 2.21 |
| **1998** | 0.27 | 0.59 | 1.73 |
| **1999** | 0.31 | 0.58 | 1.47 |
| **2000** | 0.63 | 1.18 | 2.88 |
| **2001** | 0.55 | 1.09 | 2.82 |
| **2002** | 0.52 | 0.95 | 2.81 |
| **2003** | 0.62 | 1.24 | 3.31 |
| **2004** | 0.58 | 1.38 | 4.42 |
| **2005** | 0.82 | 1.89 | 5.10 |
| **2006** | 0.70 | 1.28 | 3.91 |
| **2007** | 1.01 | 1.72 | 5.73 |
| **2008** | 1.05 | 2.50 | 6.82 |
| **2009** | 1.14 | 2.05 | 6.61 |
| **2010** | 1.92 | 4.03 | 11.70 |
| **2011** | 1.61 | 3.57 | 10.16 |
| **2012** | 1.74 | 4.16 | 13.32 |
| **2013** | 1.45 | 3.41 | 10.41 |
| **2014** | 1.92 | 4.18 | 13.11 |
| **2015** | 2.02 | 4.87 | 13.31 |
| **2016** | 2.34 | 5.34 | 16.67 |
| **2017** | 2.07 | 4.98 | 14.86 |
| **2018** | 2.53 | 6.66 | 16.89 |
| **2019** | 3.60 | 7.76 | 20.08 |
| **2020** | 3.09 | 6.73 | 18.26 |
| **2021** | 1.80 | 4.52 | 13.14 |
