# Supplementary material for: Effects of fishing restrictions on the recovery of the endangered Saimaa ringed seal (Pusa hispida saimensis) population
Source: PLoS One. 2024 Dec 5;19(12):e0311255. doi: 10.1371/journal.pone.0311255 (PMC11620628; doi:10.1371/journal.pone.0311255)
Supplement: Attachment — Submitted filename: PONE-D-24-24142_reviewer copy.pdf [file pone.0311255.s004.pdf]

PLOS ONE

Effects of fishing restrictions on the recovery of the endangered Saimaa ringed seal (Pusa hispida saimensis) population

--Manuscript Draft--

**Manuscript Number:**	PONE-D-24-24142
**Article Type:**	Research Article
**Full Title:**	Effects of fishing restrictions on the recovery of the endangered Saimaa ringed seal (Pusa hispida saimensis) population
**Short Title:**	Effects of fishing restrictions in Saimaa
**Corresponding Author:**	Pekka Jounela<br>Natural Resources Institute Finland: Luonnonvarakeskus<br>Turku, FINLAND
**Keywords:**	temporal closures, bycatch, pattern recognition, mortality, pinniped, PBR
**Abstract:**	Over the past three decades, incidental bycatch has been the single most frequent verified cause of death of the endangered Saimaa ringed seal (Pusa hispida saimensis). Spatial and temporal fishing closures have been enforced to mitigate bycatch, which is mainly caused by the gillnets of recreational fishers. In this study, we employed an array of statistical machine learning methods to recognize patterns of death and to evaluate the impacts of annual fishing closures (15th April–30th June) on the recovery of the Saimaa ringed seal population during 1991–2021. We additionally used the potential biological removal (PBR) procedure to assess bycatch sustainability. The study shows that gillnet restriction areas are reflected in the timing of juvenile bycatch mortality of the Saimaa ringed seal. In the 1990s, peak mortality occurred at the beginning of June, but as the restrictions expanded regionally in the 2000s, the peak shifted to the beginning of July. Longer temporal coverage of annual closures would have improved juvenile survival. The study also shows that estimated bycatch mortality is higher than observed: the estimated bycatch averaged approximately two unobserved bycatches per one observed bycatch. Despite the still existing bycatch mortality, a larger number of juveniles nowadays survive to the age of 15 months due to fishing closures, and the population (some 420 individuals) has increased an average 4% per year between 2017 and 2021. However, human-caused mortality limits (PBR) were exceeded by observed bycatch only, which could lead to population depletion in the long run.
**Order of Authors:**	Pekka Jounela
	Miina Auttila
	Riikka Alakoski
	Marja Niemi
	Mervi Kunnasranta
**Additional Information:**	
**Question**	**Response**
**Financial Disclosure**<br><br>Enter a financial disclosure statement that describes the sources of funding for the work included in this submission. Review the submission guidelines for detailed requirements. View published research articles from *PLOS ONE* for specific examples.	Yes

Powered by Editorial Manager® and ProduXion Manager® from Aries Systems Corporation

This statement is required for submission and **will appear in the published article** if the submission is accepted. Please make sure it is accurate.<br><br>**Funded studies**<br>Enter a statement with the following details:<br>• Initials of the authors who received each award<br>• Grant numbers awarded to each author<br>• The full name of each funder<br>• URL of each funder website<br>• Did the sponsors or funders play any role in the study design, data collection and analysis, decision to publish, or preparation of the manuscript?<br><br>Did you receive funding for this work?	
Please add funding details.<br>as follow-up to "**Financial Disclosure**<br><br>Enter a financial disclosure statement that describes the sources of funding for the work included in this submission. Review the submission guidelines for detailed requirements. View published research articles from *PLOS ONE* for specific examples.<br><br>This statement is required for submission and **will appear in the published article** if the submission is accepted. Please make sure it is accurate.<br><br>**Funded studies**<br>Enter a statement with the following details:<br>• Initials of the authors who received each award<br>• Grant numbers awarded to each author<br>• The full name of each funder<br>• URL of each funder website<br>• Did the sponsors or funders play any role in the study design, data collection and analysis, decision to publish, or preparation of the manuscript?	This work was funded by the Ministry of Agriculture and Forestry of Finland and the Our Saimaa Seal LIFE Programme of the European Commission (LIFE19NAT/FI/000832).

Did you receive funding for this work?"	
Please select the country of your main research funder (please select carefully as in some cases this is used in fee calculation).<br>as follow-up to "**Financial Disclosure**<br><br>Enter a financial disclosure statement that describes the sources of funding for the work included in this submission. Review the submission guidelines for detailed requirements. View published research articles from *PLOS ONE* for specific examples.<br><br>This statement is required for submission and **will appear in the published article** if the submission is accepted. Please make sure it is accurate.<br><br>**Funded studies**<br>Enter a statement with the following details:<br>• Initials of the authors who received each award<br>• Grant numbers awarded to each author<br>• The full name of each funder<br>• URL of each funder website<br>• Did the sponsors or funders play any role in the study design, data collection and analysis, decision to publish, or preparation of the manuscript?<br><br>Did you receive funding for this work?"	FINLAND - FI
**Competing Interests**<br><br>Use the instructions below to enter a competing interest statement for this submission. On behalf of all authors, disclose any competing interests that could be perceived to bias this work—acknowledging all financial support and any other relevant financial or non-financial competing interests.<br><br>This statement is required for submission and **will appear in the published article** if	The authors have declared that no competing interests exist.

the submission is accepted. Please make sure it is accurate and that any funding sources listed in your Funding Information later in the submission form are also declared in your Financial Disclosure statement.<br><br>View published research articles from *PLOS ONE* for specific examples.<br><br>**NO authors have competing interests**<br><br>Enter: *The authors have declared that no competing interests exist.*<br><br>**Authors with competing interests**<br><br>Enter competing interest details beginning with this statement:<br><br>*I have read the journal's policy and the authors of this manuscript have the following competing interests: [insert competing interests here]*<br><br>* typeset	
**Ethics Statement**<br><br>Enter an ethics statement for this submission. This statement is required if the study involved:<br><br>• Human participants<br>• Human specimens or tissue<br>• Vertebrate animals or cephalopods<br>• Vertebrate embryos or tissues<br>• Field research<br><br>Write "N/A" if the submission does not require an ethics statement.<br><br>General guidance is provided below. Consult the submission guidelines for detailed instructions. **Make sure that all information entered here is included in the Methods section of the manuscript.**	N/A

**Format for specific study types**<br><br>**Human Subject Research (involving human participants and/or tissue)**<br>• Give the name of the institutional review board or ethics committee that approved the study<br>• Include the approval number and/or a statement indicating approval of this research<br>• Indicate the form of consent obtained (written/oral) or the reason that consent was not obtained (e.g. the data were analyzed anonymously)<br><br>**Animal Research (involving vertebrate animals, embryos or tissues)**<br>• Provide the name of the Institutional Animal Care and Use Committee (IACUC) or other relevant ethics board that reviewed the study protocol, and indicate whether they approved this research or granted a formal waiver of ethical approval<br>• Include an approval number if one was obtained<br>• If the study involved *non-human primates*, add *additional details* about animal welfare and steps taken to ameliorate suffering<br>• If anesthesia, euthanasia, or any kind of animal sacrifice is part of the study, include briefly which substances and/or methods were applied<br><br>**Field Research**<br><br>Include the following details if this study involves the collection of plant, animal, or other materials from a natural setting:<br>• Field permit number<br>• Name of the institution or relevant body that granted permission	
**Data Availability**<br><br>Authors are required to make all data underlying the findings described fully available, without restriction, and from the time of publication. PLOS allows rare exceptions to address legal and ethical concerns. See the PLOS Data Policy and FAQ for detailed information.	Yes - all data are fully available without restriction

A Data Availability Statement describing where the data can be found is required at submission. Your answers to this question constitute the Data Availability Statement and **will be published in the article**, if accepted.<br><br>**Important:** Stating 'data available on request from the author' is not sufficient. If your data are only available upon request, select 'No' for the first question and explain your exceptional situation in the text box.<br><br>Do the authors confirm that all data underlying the findings described in their manuscript are fully available without restriction?	
**Describe where the data may be found in full sentences. If you are copying our sample text, replace any instances of XXX with the appropriate details.**<br><br>• If the data are **held or will be held in a public repository**, include URLs, accession numbers or DOIs. If this information will only be available after acceptance, indicate this by ticking the box below. For example: *All XXX files are available from the XXX database (accession number(s) XXX, XXX.*).<br>• If the data are all contained **within the manuscript and/or Supporting Information files**, enter the following: *All relevant data are within the manuscript and its Supporting Information files.*<br>• If neither of these applies but you are able to provide **details of access elsewhere**, with or without limitations, please do so. For example:<br><br>*Data cannot be shared publicly because of [XXX]. Data are available from the XXX Institutional Data Access / Ethics Committee (contact via XXX) for researchers who meet the criteria for access to confidential data.*<br><br>*The data underlying the results presented in the study are available from (include the name of the third party*	The data is available upon reasonable request.

and contact information or URL).<br>• This text is appropriate if the data are owned by a third party and authors do not have permission to share the data.<br><br>* typeset	
Additional data availability information:	

Powered by Editorial Manager® and ProduXion Manager® from Aries Systems Corporation

Manuscript

Click here to access/download;Manuscript;Manuscript_Bycatch_13thJune20

Effects of fishing restrictions on the recovery of the endangered Saimaa ringed seal (*Pusa hispida saimensis*) population

Pekka Jounela[1*], Miina Auttila[2], Riikka Alakoski[2], Marja Niemi[3], Mervi Kunnasranta[3,4]

[1]Natural Resources Institute Finland, 20520 Turku, Finland

[2]Metsähallitus, Parks & Wildlife Finland, 57130 Savonlinna, Finland

[3]University of Eastern Finland, Department of Environmental and Biological Sciences, 80101 Joensuu, Finland

[4]Natural Resources Institute Finland, 80110 Joensuu, Finland

*Corresponding author: Pekka Jounela (email: pekka.jounela@luke.fi)

All authors had a major contribution to this work. PJ was also responsible for applying statistical analyses.

Abstract

Over the past three decades, incidental bycatch has been the single most frequent verified cause of death of the endangered Saimaa ringed seal (*Pusa hispida saimensis*). Spatial and temporal fishing closures have been enforced to mitigate bycatch, which is mainly caused by the gillnets of recreational fishers. In this study, we employed an array of statistical machine learning methods to recognize patterns of death and to evaluate the impacts of annual fishing closures

(15th April–30th June) on the recovery of the Saimaa ringed seal population during 1991–2021. We additionally used the potential biological removal (PBR) procedure to assess bycatch sustainability. The study shows that gillnet restriction areas are reflected in the timing of juvenile bycatch mortality of the Saimaa ringed seal. In the 1990s, peak mortality occurred at the beginning of June, but as the restrictions expanded regionally in the 2000s, the peak shifted to the beginning of July. Longer temporal coverage of annual closures would have improved juvenile survival. The study also shows that estimated bycatch mortality is higher than observed: the estimated bycatch averaged approximately two unobserved bycatches per one observed bycatch. Despite the still existing bycatch mortality, a larger number of juveniles nowadays survive to the age of 15 months due to fishing closures, and the population (some 420 individuals) has increased an average 4% per year between 2017 and 2021. However, human-caused mortality limits (PBR) were exceeded by observed bycatch only, which could lead to population depletion in the long run.

Key words: temporal closures, bycatch, pattern recognition, mortality, pinniped, PBR

1. Introduction

The unintended capture of nontarget species, or "bycatch," is one of the main direct human-induced mortality causes of marine megafauna. Bycatch can cause injury or even death to the animals involved [1]. Marine mammals can become entangled in various fishing gears, such as trawls, purse seines, longlines, gillnets, and pot/trap fisheries, or they may be accidentally struck by fishing vessels [2, 3, 4, 5, 6]. Seal–fishery interactions are a major conservation concern also for several pinniped species globally [7], and incidental bycatch has been identified as a major threat for the most endangered rare seal populations [6, 7, 8, 9, 10, 11].

The landlocked Saimaa ringed seal (*Pusa hispida saimensis*) population in Finland has suffered from bycatch mortality, which has been implicated ~~as a major driver in the decline of this endangered endemic seal [8], and bycatch has been identified~~ as a primary threat to the Saimaa ringed seal population [7]. After weaning, bycatch is shown to be the dominant mortality factor for juveniles [6, 8], and the problem is still acute despite active conservation efforts during past decades [12, 13]. The negative consequences of bycatch mortality for the population's long-term survival have been highlighted during a time in which this endangered subspecies is increasingly suffering from the changing climate [14]. Saimaa ringed seals are dependent on ice and snow as a breeding habitat and are, therefore, suffering from direct negative effects of climatic warming. Pups are born in subnivean lairs in February–March, and nursing lasts 7–12 weeks [15, 16, 17, 18]. Saimaa ringed seals ~~are suggested to~~ display high breeding site fidelity, and females are shown to exhibit at least mutual avoidance during the breeding period [19]. Perinatal mortality is relatively high and is suggested to be related to poor breeding habitat conditions due to mild winters [20] or inbreeding pressure [21]. Moulting occurs after the breeding season in May–June, and the seals use terrestrial platforms for moulting [22].

Globally, gillnets are one of the most lethal fishing gears for pinnipeds [6]. This is also seen in Lake Saimaa, where the majority of observed seal bycatch mortality is caused by gillnets [26], mainly owned by recreational fishers [8]. Only 30–40 commercial fishers work in Lake Saimaa, but the exact volume of the larger-scale recreational fishery is not known. Estimates vary between 49 000–400 000 recreational fishers, a large proportion of which use passive gear such as gillnets and/or wire traps [23, 24, 25, 26]. The vendace (*Coregonus albula*) is a target species of greater economic importance for commercial fishing in Lake Saimaa. It is mostly caught with seal-safer gear, such as trawls and seines, but also with gillnets in some places [27]. The other economically important target species, such as pike perch (*Sander lucioperca*), perch (*Perca fluviatilis*), and pike (*Esox lucius*), are typically caught with gillnets. These species are also the most common catches for recreational fishers [25, 28]. In general, a significant part of fishing is conducted using passive gear, and 36% of all recreational fishing catches in Finland in 2020 were made using gillnets [29].

Pinniped bycatch mitigation methods are typically related to modifications of fishing gear type and practice and to fishery closures [4, 30, 31]. In Lake Saimaa, these measures have included the introduction of gear modifications, such as thinner gillnet materials, fish traps, and fyke net models with bars to keep seals out, and prohibiting the use of longlines and fish-baited fishing hooks. In addition, education and outreach programmes have helped raise awareness among fishers and the public on the impacts of bycatch and the importance of responsible fishing practices [14]. The implementations of temporal and spatial fishing closures have been the key conservation measures since the 1990s [8]. The most dangerous fishing methods for seals are banned year-round in the main Saimaa ringed seal distribution area by the Government Decree on Fishing Restrictions (374/2021). In addition, springtime fishing area closures for gillnet

fishing have been enforced from 15th April to 30th June, which is suggested to be the most critical period for pup survival [8]. In 1991, springtime spatial closures only covered some 18% of birth lair sites, but since the end of the 2010s this coverage has been around 95% [13]. However, impacts of the long-term temporal and spatial fishing restrictions on the population growth rate have not been evaluated, although the seal population has slowly recovered during the past decades from the strongest decline [14]. Increased measures due to seal conservation, especially fishing restrictions, have concurrently increased local socioeconomic conflicts [24].

Fishing in Lake Saimaa is not managed based on biological reference points that inform fisheries managers of the limits beyond which the state of a fishery and/or a resource is undesirable. Limits for allowable human-caused mortality can be assessed using the potential biological removal approach (PBR; [32]). In general, the PBR approach is a reference point method originally developed to assess human-caused mortality limits of marine mammal populations [33]. The maximum net productivity level (MNPL) underpins PBR and is nearly identical to the concept of maximum sustainable yield (MSY) often used in fish stock assessments [34]. In the PBR approach, MNPL is often considered to be 50% of the environment's carrying capacity (K), and the optimum sustainable population (OSP) is defined as being between K and MNLP. Marine mammal populations should not be permitted to diminish below the OSP level. If this happens, management actions may be taken to recover the depleted population. The U.S. Marine Mammal Protection Act (MMPA) requires calculating PBR reference points for all stocks in assessment reports [35]. Estimating bycatch limits in terms of PBR reference points would also improve conservation planning regarding the Saimaa ringed seal population.

This study presents the key findings of an analysis conducted to estimate the bycatch mortality of the Saimaa ringed seal caused by fishing for 1991–2021. The aim is to evaluate the impact

of the past and proposed extended temporal fishing restrictions on the recovery of the seal population. The specific objectives are 1) to estimate the timing of the bycatch mortality related to the spatial coverage of the fishing closures, 2) to study how the extended temporal coverage of annual restrictions would have improved pup survival, 3) to estimate the magnitude of unobserved bycatch mortality, 4) to estimate population size, and 5) to use the PBR approach for calculating whether observed and estimated bycatch levels are consistent with reaching the target growth rate (3−6% per year using a five-year moving average to reduce the impact of random and natural annual variation, Ministry of the Environment 2023) of the Saimaa ringed seal. We employed an age group-specific population model and an array of machine learning techniques to recognize patterns of deaths that are too complex to be perceptible by human researchers employing standard statistical tools.

2. Materials and methods

2.1. Study area

Lake Saimaa (61° 05−62° 36’ N, 27° 15−30° 00’ E) is a labyrinthic freshwater system in eastern Finland. It is approximately 180 km long and 140 km wide, and the study area covers the Saimaa ringed seal distribution (Figure 1). Metsähallitus Parks & Wildlife Finland (MHPWF) is a public authority responsible for Saimaa ringed seal population monitoring and most of the conservation actions. Data on seal mortality, population estimates, lair sites, and numbers of pups born in 1991−2021 used in the analyses were obtained from the MHPWF database [36]. In addition, a spatial coverage estimate (%) of the springtime (15th April–30th June) fishing area closures for gillnet fishing in relation to birth lairs was used.

2.2. Data on mortality

Since the 1990s, all dead Saimaa ringed seals that have been reported to MHPWF have been collected and examined. The mortality data used in our study, including causes of death (Table 1), location, date, and age, were based on 554 carcasses examined between 1991 and 2021. The cementum layers in the lower canine teeth were counted to determine seal ages (apart from pups with lanugo hair; [ ]). In addition, 98 individuals in the dataset were not included in this study due to lack of information on their exact age. A wildlife pathologist identified the causes of death, or field verification was obtained for instances of entanglement in fishing gear. In addition to those known to have died as bycatch (found in fishing gear), suffocations detected by a wildlife pathologist were also defined as observed bycatch mortality (Table 1). Hereafter, we define the word “observed” as information that exists in the MHPWF database as opposed to “unobserved” information that does not exist in any database. A model result is referred to as “estimated”, and unobserved information is calculated by subtracting from the estimated observed information (i.e. estimated = observed + unobserved).

Table 1. Causes of death frequencies and four main cause of death groups in the Saimaa ringed seal mortality data.

Cause of death, all age groups	Frequency	Four main cause of death groups	Frequency
Stillborn	63	Stillborn	63
Predated lair deaths	5	Lair deaths	108
Other lair deaths	97		
Unweaned other deaths	6		
Fishing (self-reported)	142	Fishing	163
Suffocation	21		
Natural	26	Other	220
Unknown	186		
Accidental	1		
Cancer	2		
Boat collision	4		
Violence	1		
Sum	554		554

2.3. Data on pups and birth lairs

As Saimaa ringed seals give birth to a single pup, birth lairs are used to estimate the annual number of pups born since the 1990s [8]. Information on lair sites and pup numbers are based on lair censuses and on lair site diving surveys from 2014 onwards, conducted annually throughout Lake Saimaa (20, 37). Observed pup numbers are based on confirmed evidence of birth (placenta, lanugo hair, alive or dead pup). However, not all pups are found with absolute certainty, which is why the number of born pups is estimated using a three-year average in areas where the census has not been comprehensive during the current year. Therefore, birth lair data included both observed and estimated numbers of pups and locations of birth lairs during 1991−2021.

2.4. Statistical methods

2.4.1. Model structure

Bycatch was estimated using an age group-specific population model in Rapidminer Studio Enterprise software (version 9.10.011; www.rapidminer.com, [38], see also [13]). We analysed pup survival between 1991 and 2021, first as pups from ages 0 to 15 months and then as adults up to 30 years of age in 1-year intervals. The total mortality rate, i.e. the number of all deaths at age at x, was computed separately for individuals aged less than or equal to 15 months and older. A two-stage approach was employed here because most carcasses (68%) were of individuals less than 15 months old [13], and for this reason the annual change in the adult population size was estimated starting with the survivors in the 15-month age group. Due to the low annual number of samples in the observed carcass data (5.4 individuals per year on average), over 15-month-old seals were counted as one joint survival estimate for 1991–2021. The population size estimate included 30 age groups (from 1 year to 30 years). The starting

assumption was that the 1990 population size of this study equalled that of MHPWF (mean 189, range 164–210; [26].

The starting data used in the analysis was the total number of annually born pups minus the number of observed deaths at age 0 months (*Nage0*). However, not all deaths are observed during the first two-week period, and hence the total number of 0.5-month-old seals (*Nage0.5*) at the end of the first two-week period (0 -> 0.5 months) was estimated as:

$$N_{age0,5} = N_{age0} - N_{age0} * exp^{\left(-\frac{b}{2}\right)} * Pr_{age0} \quad (1)$$

where *b* is the slope parameter of the linear regression line directly proportional to the total mortality rate assessed by plotting the carcass frequencies (ln + 1 transformed) by month and year (also known as the slope parameter of the 'catch curve' mortality estimation method; [39, 40], 2 denotes catch curve mortality estimation in 2-week age periods, and Pr denotes the k-nearest neighbour model (k-NN; [41]) -based proportion (range: 0, …, 1) of deaths during the first 2 weeks of age. Parameter k of the k-NN model was optimized using 10-fold cross validation [42]. The catch curve mortality estimation method infers total mortality rates based on the changes in (carcass) age distribution. It is a widely used technique in fisheries science, especially with data-poor fish stock assessments. The catch curve mortality estimation method assumes that mortality is the only reason for the decrease in individual numbers across the ages of the population i.e., there is no emigration or immigration in the study area. Note here that the catch curve mortality estimation method assumes linear age distributions, and hence we also apply nonlinear k-NN-based proportions of deaths in 2-week periods to recognize more realistic nonlinear mortality patterns. Bycatch has never been observed during the first 2-week

age period and was hence assumed to be zero. Thereafter, bycatch C at age 0.5 – 15 months during the closed and open gillnet seasons and area was calculated as:

$$C_{age} = N_{age} - N_{age} * exp^{\left(-\frac{b}{2}\right)} * Pr_{age} * P\left(C_{age}\right) * O_{age} \quad (2)$$

where *P(Cage)* denotes confidence (sometimes also referred to as probability; range 0, …, 1) of bycatch death with respect to all other causes of deaths at age 0.5 – 15 months and *Oage* denotes the proportion (range 0, …, 1) of open gillnet fishing area at age. The remaining deaths at age 0.5 – 15 months caused by other reasons occurred in all (open and closed) areas.

2.4.2. Causes of death estimation

The confidence distributions for the causes of death in terms of age, year, and water area were computed using four main cause of death groups: stillborn, which consisted of pups dead at birth; lair death, which consisted of mostly unverified causes of death of unweaned pups aged 0−2 months (multiple, mostly unverified causes of death); fishing, which consisted of observed bycaught seals aged 0−35 years; and other, which consisted of seals older than 2 months that died of natural or unknown causes.

The reason for not using all the original cause of death groups (N=12, Table 1) was to minimize the computational complexity of the algorithm. On the other hand, more than two cause of death groups (fishing and other than fishing) were used in the model because our trial model runs showed better 10-fold cross-validation performance of the model. Cause of death patterns were recognized using a random forest (RF) model [43]. In general, RF is a machine learning method that consists of building an ensemble [44] classifier of decision trees produced from bagging (bootstrap aggregation; see [45]) and a randomized variant of the tree induction

algorithm. The four parameters of the RF model, i.e. number of trees, maximal depth, criterion (accuracy, gini index, information gain, gain ratio), and confidence level used for the pessimistic error calculation of pruning, were optimized using 10-fold cross-validation [42]. The assumption is equal to the detection probabilities of carcasses across ages, years, locations, and causes of death.

2.4.3. Simulated change in fishing restrictions

We also simulated momentary survival rate if (and only if) the start of gillnet fishing in 1991–2021 had been the 1st of August, the 1st of October, or the ban on gillnet fishing had been year-round. Currently, fishing restrictions reach until the end of June. There is ongoing debate on continuing these restrictions, and on using month as the temporal unit. We therefore also use months as an example. The word momentary here means that simulated change in survival does not consider the possible long-term effects after the change, such as a possible increase in recruitment in upcoming years.

2.4.4. Potential biological removal

The PBR approach [32] was used for calculating whether observed and estimated bycatch levels are consistent with reaching the Saimaa ringed seal target growth rate. The PBR was calculated as:

$$PBR = N_{Min} \frac{1}{2} R_{Max} F_R \qquad (3)$$

where NMIN is the 20th percentile of a log-normal distribution based on an estimate of the number of animals in a stock (which is equivalent to the lower limit of a 60% two-tailed confidence interval (CI)), ½ RMAX is one-half of the maximum theoretical or estimated net productivity rate of the stock as a small population size, and FR is a recovery factor between

0.1 and 1. That is, for all adult cohorts in all years, we apply a lower limit of a 60% CI survival estimate. The input values used here were default ones used with endangered pinniped populations RMAX = 0.12 and FR = 0.1 [46].

3. Results

In 1991–2021, fishing was responsible for 26% (n = 142 / 554; Table 1) of the observed (self-reported) mortality and 90% (n = 128) of these bycaught seals were entangled in gillnets.

3.1. Temporal distribution of bycatch mortality

The distribution of estimated bycatch mortality during the first 15 months of a Saimaa ringed seal's life showed two peaks over the past thirty years (Figure 2a). The estimated first bycatch peak in the 1990s occurred at the beginning of June, when pups were three months old. Since the early 2000s, the first estimated bycatch peak shifted to the beginning of July (Figure 2a). However, temporal shifts did not occur in the bycatch peak at the beginning of March, when the ringed seals born the previous year were approximately one year of age.

3.2. Delayed start date of gillnet fishing

A longer temporal closure for gillnet fishing would improve juvenile survival. Postponing the start of gillnet fishing to early August would have increased juvenile survival (0–15 months) by 1.8 individuals in 2021 (Figures 2b and 3, S2). Similar momentary estimates for gillnet fishing starting in early October and a year-round ban on gillnet fishing in 2021 would have increased survival by 4.5 and 13.1 individuals, respectively (Figures 2c, 2d and 3). However, postponing the start of gillnet fishing to the beginning of August or October would have increased the bycatch numbers of winter fishing (Figures 2c and 2d). Nevertheless, winter and autumn fishing would not eliminate the benefit (increase in survival) that would result from

postponing the start of gillnet fishing by 1–3 months (Figures 2a–d and 3). Note that the estimated increases in survival are instantaneous estimates, which means that the calculation does not consider the possible long-term (multi-year) cumulative effects of the change.

3.3. Estimated bycatch numbers

The bycatch estimated in this study averaged 1.8 unobserved fishing deaths per one observed. The number of observed fishing-related deaths has increased somewhat compared to the 1990s. The observed annual bycatch averaged 4.44 individuals in the 1990s, 6 individuals in the 2000s, and 5.25 individuals in 2010–2021 (Table 2). Over the same period, the number of bycatches estimated by the model, i.e., the number of observed and unobserved deaths, has increased by 1.7-fold and the number of ringed seals that survived the first 15 months has increased by 3.1-fold. For example, the average annual estimated number of bycaught seals in 2010–2021 was 18.86 (Figure 4). Seal bycatch rate in terms of average annual estimated number of bycaught seals per average annual estimated number of survivors at age 0.5 months was 0.41 in the 1990s, 0.37 in the 2000s, and 0.32 in 2010-2021 (max 0.46 in 1993; min 0.24 in 2021; Table 2). The carcass data suggested an average 40% suffocation rate for over 0.5-month-old seals found accidentally (mostly stranded seals) in 1991–2021. During the past decade, an annual average of ~ 70 survivors in the over 0.5-month-old recruits and an increase in the population size by ~ 10 individuals suggested ~ 60 deaths, of which roughly 24 (60 x 40% = 24) individuals had suffocated (bycatch). In addition, during 1991–2000, an annual average of 33 estimated survivors in the over 0.5-month-old recruits and an estimated increase in population size by 4 individuals suggested an estimated 29 deaths, of which 11.6 (29 x 40% = 11.6) individuals had suffocated (bycatch) (see also Table 2 and Fig. 4).

Table 2. Estimated and observed bycatch, number of pups born, estimated all deaths, and estimated other deaths of seals aged 0–15 months (see also S1), estimated 15-month-old survivors, estimated population size (mean; number of individuals), and bycatch rate of seals in terms of the average annual estimated number of bycaught seals/average annual estimated number of survivors at age 0.5 months in 1991–2021. During 2017–2021, the population growth rate averaged 4%. In total, birth lair data included the observed and estimated numbers of pups (observed N = 1557, estimated N= 1778) and birth lair locations (N = 1526) during 1991−2021.

**Year**	**Est. bycatch**	**Obs. bycatch**	**Est. no. of pups born**	**Est. all deaths**	**Est. other deaths**	**Est. 15-month-old survivors**	**Est. population size**	**Est. bycatch / Est. 0.5-month-old survivors**
**1991**	9.7	3	31	16.6	6.8	11.4	189.7	0.44
**1992**	9.7	1	34	18.3	8.6	12.7	191.0	0.39
**1993**	13.0	9	36	20.0	7.0	14.0	193.5	0.46
**1994**	11.4	4	38	18.2	6.8	12.8	194.7	0.44
**1995**	10.4	4	39	21.2	10.9	14.8	197.8	0.36
**1996**	9.5	0	40	15.8	6.3	11.2	197.2	0.44
**1997**	15.7	7	44	24.6	8.9	17.4	202.9	0.45
**1998**	11.9	10	41	22.2	10.3	15.8	206.7	0.37
**1999**	10.7	2	42	23.7	13.0	16.3	210.8	0.34
**2000**	17.6	6	57	31.5	13.9	22.5	221.0	0.42
**2001**	16.3	4	56	27.4	11.1	19.6	227.8	0.43
**2002**	14.8	8	56	31.3	16.5	22.7	237.4	0.33
**2003**	16.2	8	51	27.1	10.9	19.9	243.7	0.42

**2004**	18.6	6	66	35.0	16.4	25.0	254.9	0.36
**2005**	15.7	5	51	26.7	11.0	20.3	260.8	0.41
**2006**	12.7	6	52	23.2	10.6	17.8	264.0	0.37
**2007**	12.7	6	55	26.1	13.4	20.9	270.1	0.32
**2008**	11.9	5	51	26.1	14.2	20.9	276.0	0.30
**2009**	9.5	6	44	20.5	11.0	17.5	278.2	0.31
**2010**	16.0	5	57	28.4	12.4	24.6	287.4	0.37
**2011**	13.0	4	57	24.8	11.8	21.2	292.8	0.34
**2012**	16.9	7	61	31.5	14.6	26.5	303.3	0.35
**2013**	13.7	7	62	27.9	14.2	23.1	309.9	0.31
**2014**	15.4	7	64	29.6	14.1	25.4	318.5	0.33
**2015**	15.7	5	71	33.7	18.0	28.3	329.6	0.31
**2016**	20.1	5	87	43.4	23.3	35.6	347.5	0.30
**2017**	17.7	7	83	37.3	19.6	30.7	359.7	0.31
**2018**	19.7	4	86	42.0	22.3	34.0	374.7	0.31
**2019**	22.7	6	88	46.8	24.1	40.2	395.2	0.33
**2020**	20.7	4	88	42.5	21.8	34.5	409.0	0.33
**2021**	15.9	2	90	45.2	29.3	33.8	421.6	0.24

3.4. PBR

The estimated bycatch in 2021 (15.88 individuals) was higher than the PBR estimate (2.48) for that year. In most (27/31) years the observed and in all years the estimated bycatch was higher than the estimated PBR in 2021 (Table 2). The average estimated bycatch in 1991–2021 (14.69

individuals) was 5.9 times higher than the PBR in 2021. The lower limit of a 60% two-tailed confidence interval for stock size (NLower60%) in 2021 was 414 individuals.

4. Discussion

This is the first study that investigates the effects of fishing restrictions on the endangered Saimaa ringed population over three decades. Based on our results, bycatch mortality seems to be up to three times more prominent than what is observed and causes additional mortality, especially in young seals up to 15 months of age. This creates an increased depletion risk for the population, as the present bycatch mortality rates exceed the estimated allowable human-caused mortality.

4.1. Bycatch distribution

Saimaa ringed seals are most vulnerable to bycatch mortality as juveniles, and 90% of lethal entanglements are caused by gillnets. Bycatch typically occurs in summer, but this mortality can also be seen during other seasons. Over the previous 30 years, the bycatch mortality distribution during the first 15 months of a Saimaa ringed seal's life has been two-peaked. The first peak occurred during June–July and the second peak in March. At the time of minor fishing restrictions in the 1990s, the bycatch mortality peak occurred already at the beginning of June, but as the restrictions expanded regionally in the 2000s, the peak shifted to the beginning of July. The second mortality peak in March is caused by stationary gillnets set under ice. This is somewhat contrary to the raw data, where bycatch mortality was quite similar throughout January–March. The difference may have been caused by the relatively small number of bycatches or by parametrization of the RF model. Nonetheless, wintertime gillnetting causes mortalities, because it has not been restricted in most areas. Our study indicates that the coverage of present springtime closures in the main Saimaa ringed seal distribution area may

be regionally adequate, but temporally too short. Therefore, a longer temporal closure for gillnet fishing would be an important conservation action to improve juvenile survival. For example, extending fishing restrictions until the end of July in 2021 would have saved around two seals (1.79) and a ban until the end of September around four (4.5) seals. In addition, autumn and winter fishing would not eliminate the increase in survival if gillnet fishing were postponed further until the end of July or September. Also, a total ban on gillnet fishing in Lake Saimaa has been publicly discussed, and a year-round gillnet closure would have increased survival by 13 seals in 2021.

4.2. Unobserved bycatch

Most Saimaa ringed seal bycatch mortality is unobserved. Our~~ study showed tha~~t observed bycatch mortality was roughly one-third of the estimated fishing deaths. It is widely accepted that accurate estimation of bycatch rates requires an independent observer scheme [47]~~. This i~~s ~~because~~ The observed bycatch ~~numbers~~ typically varies, and the observed numbers do not represent the actual magnitude of bycatch mortality [e.g., 4, 48]. Underreporting may also be unintentional, as small entangled marine mammals (such as seals) may free themselves from the fishing gear while it is being retrieved, unseen by fishers [49]. Bycatch reporting for seals has been ~~requested, and after 2016,~~ mandated in Lake Saimaa since 2016 (62 §, Fishing Act 379/2015; Finnish legislation available at www.finlex.fi). ~~However, these~~ r Reporting rates are frequently low, however, and using these data usually leads to negatively biased bycatch rate estimates [50, 51, 52]. In the present study, the average estimated bycatch level was based on both self-reported and suffocated seals.

The estimated bycatch of the present study could be biased because it is statistically confounded with estimated other (natural and unknown) deaths of over 0.5-month-old seals. That is, for seals over 0.5 months of ag~~e~~, estimated other mortality fact~~ors fac ors of for seals over 0.5-month old seals~~ affect the estimated bycatch ~~of over 0.5-month aged seals,~~ and vice versa. Another potential bias source is the

inability to reliably quantify the latent causes of bycatch, such as sudden changes in fishing effort. Overall, we have little information on the fishing effort in Lake Saimaa, and the incidental bycatch reporting rate may vary between seasons and years.

4.3. Depletion risk

Although the Saimaa ringed seal population is slowly growing, it is good to observe that the small population has an extremely narrow gene pool [21, 53, 54], and it is seriously threatened by habitat loss due to human activities and climate change [14, 20, 55]. All these factors increase population vulnerability to decline when anthropogenic influences, such as bycatch mortality, increase total mortality levels. Accordingly, our PBR results suggest an increased risk of depletion, and, therefore, conservation actions, e.g., longer gillnet closures, should be implemented. In most years, even the observed bycatch was higher than the estimated PBR. In addition, when estimated bycatch numbers are included, annual human-induced mortality was 5.9 times larger than the estimated PBR in 2021. According to Wade [32], a marine mammal population is at risk of going extinct if the human-caused mortality estimate exceeds its PBR. Even if the extra conservative Fr value 0.1 were not selected and an Fr of 0.5 were selected instead, the PBR for Saimaa seals would still be exceeded.

While habitat availability and quality are reduced by other indirect causes, bycatch influences population trends. Unintentional b Bycatch directly increases the risk of extinction, . Management actions would not be initiated at once due to an inability to detect a major decline in abundance. [illegible]he issue becomes more severe for smaller populations, as the accuracy of estimating their abundance decreases along with their population size [56]. Consequently, a small population experiencing a decline could gom e m xtinct before any significant decline is detected [32]. Therefore, it is mportant to no,mtic me that altmhorugh the population trend has been growing during

2017–2021 by an average 4% due to active measures, bycatch mortality is an ongoing risk for the long-term survival of the small Saimaa ringed seal population.

5. Conclusions

Globally, incidental bycatch in marine fisheries is typically related to commercial fisheries (e.g., 31, 49¸ 57), however, ~~studies have also indicated that~~ the ecological impacts of small-scale fisheries can be severe for many species groups [e.g., 58, 59, 60]. ~~This is also the case in~~ In Lake Saimaa, ~~where~~ recreational fishing is the major cause for ringed seal bycatch mortality. ~~Additionally~~, t The gillnet has, by far, been the most lethal gear type for Saimaa ringed seals, ~~with a~~ accounting for 90% of the incidental bycatch ~~share in Lake Saimaa~~. The lethality of gillnets has also been shown in the Baltic Sea [57] and worldwide [4]. Also, other endangered species are bycaught in gillnets in Lake Saimaa, such as the landlocked Atlantic salmon (*Salmo salar m. sebago*), the Arctic charr (*Salvelinus alpinus*), and the wild trout (*Salmo trutta*). For these reasons, more effective gillnet fishing restrictions are needed in Lake Saimaa.

Acknowledgements

We thank wildlife pathologist Marja Isomursu (Finnish Food Safety Authority) for performing the seal carcass necropsies and Mairi Young for comments on the manuscript. The material reflects the views of the authors; neither the European Commission, the CINEA, nor the Ministry of Agriculture and Forestry of Finland is responsible for any use that may be made of the information it contains.

References

1 Taylor N, Authier M, Bangac R, Genu M, Macleod K, Gilles A (2022). Marine Mammal By-catch. In: OSPAR, 2023: The 2023 Quality Status Report for the Northeast Atlantic. OSPAR Commission, London. Available at: https://oap.ospar.org/en/ospar-assessments/quality-status-reports/qsr-2023/indicator-assessments/marine-mammal-bycatch

2 Lewison RL, Crowder LB, Read AJ, Freeman SA (2004) Understanding impacts of fisheries bycatch on marine megafauna. Trends ecol evol 19:598–604

3 Lewison RL, Crowder LB, Wallace BP, Moore JE, Cox T, Zydelis R, McDonald S, DiMatteo A, Dunn DC, Kot CY, Bjorkland R, Kelez S, Soykan C, Stewart KR, Sims M, Boustany A, Read AJ, Halpin P, Nichols WJ, Safina C (2014) Global patterns of marine mammal, seabird, and sea turtle bycatch reveal taxa-specific and cumulative megafauna hotspots. Proc Natl Acad Sci USA 111:5271–5276

4 Read A J, Drinker P, Northridge S (2006) Bycatch of marine mammals in U.S. and global fisheries. Conserv Biol 20: 163–169

5 Osinga N, Ferdous MS, Morick D, Hartmann, MG, Ulloa JA, Vedder L, de Haes HU, Brakefield PM, Osterhaus ADME, Kuiken T (2012) Patterns of stranding and mortality in common seals (*Phoca vitulina*) and grey seals (*Halichoerus grypus*) in The Netherlands between 1979 and 2008. J comp pathol 147:550–565

6 Reeves RR, McClellan K, Werner TB (2013) Marine mammal bycatch in gillnet and other entangling net fisheries, 1990 to 2011. Endang Species Res 20:71–97

7 Kovacs KM, Aguilar A, Aurioles D, Burkanov V and others (2012) Global threats to pinnipeds. Mar Mamm Sci 28:414–436.

8 Sipilä T (2003) Conservation biology of Saimaa ringed seal (Phoca hispida saimensis) with reference to other European seal Populations. PhD thesis, University of Helsinki, Finland

9 Goldsworthy SD, Page B (2007) A risk-assessment approach to evaluating the significance of seal bycatch in two Australian fisheries. Biol Conserv 139: 269–285

10 Karamanlidis AA, Androukaki E, Adamantopoulou S, Chatzispyrou A, Johnson WM and others (2008) Assessing accidental entanglement as a threat to the Mediterranean monk seal Monachus monachus. Endang Species Res 5: 205–213

11 Dmitrieva L, Kondakov AA, Oleynikov E, Kydyrmanov A, Karamendin K, Kasimbekov Y, Baimukanov M, Wilson S, Goodman SJ (2013) Assessment of Caspian seal by-catch in an illegal fishery using an interview-based approach. PloS one 8:.e67074

12 Niemi M, Auttila M, Viljanen M, Kunnasranta M (2013) Home range, survival and dispersal of endangered Saimaa ringed seal pups: implications for conservation. Mar Mamm Sci 29:1–13

13 Jounela P, Sipilä T, Koskela J, Tiilikainen R, Auttila M, Niemi M, Kunnasranta M (2019) Incidental bycatch mortality and fishing restrictions: impacts on juvenile survival in the

Endangered Saimaa ringed seal *Pusa hispida saimensis*. Endang Species Res 38:91–99 doi: 10.3354/esr00939

14 Kunnasranta M, Niemi M, Auttila M, Valtonen M, Kammonen J, Nyman T (2021) Sealed in a lake - biology and conservation of the endangered Saimaa ringed seal: a review. Biol Conserv 253:108908 https://doi.org/10.1016/j.biocon.2020.108908

15 Helle E, Hyvärinen H, Sipilä T (1984) Breeding habitat and lair structure of the Saimaa ringed seal Phoca hispida saimensis Nordq. in Finland. Acta Zool Fenn 172:125–127

16 Sipilä T (1990) Lair structure and breeding habitat of the Saimaa ringed seal (Phoca hispida saimensis Nordq.) in Finland. Finn Game Res 47:11–20

17 Sipilä T, Hyvärinen H (1998) Status and biology of Saimaa (Phoca hispida saimensis) and Ladoga (Phoca hispida ladogensis) ringed seals. In: Heide-Jørgensen MP, Lydersen C (Eds.), Ringed Seals in the North Atlantic. NAMMCO, Tromsø, Norway, pp. 83–99.

18 Niemi M (2013) Behavioural ecology of the Saimaa ringed seal — implications for conservation. PhD thesis, University of Eastern Finland, Joensuu, Finland.

19 Niemi M, Liukkonen L, Koivuniemi M, Auttila M, Rautio A, Kunnasranta M (2019) Winter behavior of Saimaa ringed seals: Non-overlapping core areas as indicators of avoidance in breeding females. PLoS ONE 14:e0210266

20 Auttila M, Niemi M, Skrzypczak T, Viljanen M, Kunnasranta M (2014) Estimating and mitigating perinatal mortality of the endangered Saimaa ringed seal (*Phoca hispida saimensis*) in a changing climate. Ann Zool Fenn 51: 526−534

21 Löytynoja A, Rastas P, Valtonen M, Kammonen J, Holm L, Olsen MT, Paulin L, Jernvall J, Auvinen P (2023) Fragmented habitat compensates for the adverse effects of genetic bottleneck. Curr Biol33:1009–18

22 Niemi M, Nykänen M, Biard V, Kurkilahti M, Kunnasranta M. (2022) Molting phenology of a lacustrine ringed seal, Pusa hispida saimensis. Ecol Evol 12:e9248

23 Ratamäki O, Salmi P (2015) The most contested in Finland: Large carnivores and the Saimaa ringed seal – Challenges of socio-ecological rhythms and their practical implications. Eur Countrys 7 1–15 DOI: 10.1515/euco-2015-0001

24 Salmi P, Sipponen M (2017) Cultural strengths and governance challenges of a northern inland fishery. In: Song AM, Bower SD, Onyango P, Cooke SJ, Chuenpagdee R (eds) Inter-sectoral governance of inland fisheries. Too Big To Ignore-WorldFish, St. John's,Newfoundland, Canada p 84–96

25 Kolari I, Eskelinen P, Auvinen H, Hirvonen E, Jounela P, Moilanen P (2018) Vapaa-ajan kalastus Saimaalla 2014–2015. Luonnonvara- ja biotalouden tutkimus 32 / 2018, Luonnonvarakeskus, Helsinki, Finland p 36 (in Finnish)

26 Working group on Saimaa ringed seal and fishing (2021). Final report of the working group on Saimaa ringed seal and fishing. In: Publications of the Ministry of Agriculture and Forestry 2021:1 Helsinki, Finland p.60 http://urn.fi/URN:ISBN:978-952-366-393-0 (in Finnish with English summary)

27 Salmi J, Salmi P, Hirvonen E (2011) Ammattikalastus saimaannorppa-alueella. RKTL:n työraportteja 16/2011. Riista- ja kalatalouden tutkimuslaitos, Helsinki. (in Finnish)

28 Seppänen E, Toivonen A-L, Kurkilahti M, Moilanen P (2011) Suomi kalastaa 2009. Riista- ja Kalatalous. Tutkimuksia ja selvityksiä 1 / 2011.(in Finnish)

29 OSF (Official Statistics of Finland) (2022) Recreational fishing 2020 [web publication]. Helsinki: Natural Resources Institute Finland [referred: 23.5.2023]. Access method: https://www.luke.fi/en/statistics/recreational-fishing/recreational-fishing-2020

30 Werner T, Kraus S, Read A, Zollett E (2006) Fishing techniques to reduce the bycatch of threatened marine animals. Mar Technol Soc J 40:50–68 https://doi.org/10.4031/002533206787353204

31 Hamilton S, Baker GB (2019a) Technical mitigation to reduce marine mammal bycatch and entanglement in commercial fishing gear: lessons learnt and future directions. Rev Fish Biol Fish 29:223–247

32 Wade PR (1998) Calculating limits to the allowable human-caused mortality of cetaceans and pinnipeds. Mar Mamm Sci 14:1–37

33 Punt AE, Siple M, Francis TB, Hammond PS, Heinemann D, Long KJ, Moore JE, Sepúlveda M, Reeves RR, Sigurðsson GM, Vikingsson G. (2020) Robustness of potential biological removal to monitoring, environmental, and management uncertainties. ICES JMar Sci 77:2491–2507

34 Wade PR, Long KJ, Francis TB, Punt AE, Hammond PS, Dennis H et al. (2021) Best practices for assessing and managing bycatch of marine mammals. Front Mar Sci 8: 757330 doi: 10.3389/fmars.2021.757330

35 NMFS (2023) Guidelines for Preparing Stock Assessment Reports Pursuant to the Marine Mammal Protection Act. Available at: https://www.fisheries.noaa.gov/national/marine-mammal-protection/marine-mammal-protection-act-policies-guidance-and-regulations

36 Ministry of the Environment (2023) Conservation strategy and action plan of the Saimaa ringed seal 2022 (Saimaannorpan suojelun strategia ja toimenpidesuunnitelma 2022). In: Letter (VN/916/2023-YM-1) of the Ministry of the Environment endorsing the conservation strategy and action plan of the Saimaa ringed seal (annex to the letter). Available at: https://api.hankeikkuna.fi/asiakirjat/32b01c16-8d1c-45e2-aa71-0d8f8f52d67f/2333ea1d-281d-409d-b4d3-5f1e9dad3a46/STRATEGIA_20230612071740.pdf (in Finnish)

37 Sipilä T, Helle E, Hyvärinen H (1990) Distribution, population size and reproductivity of the Saimaa ringed seal (*Phoca hispida saimensis* Nordq.) in Finland, 1980-84. Finn Game Res 47:310

38 Mierswa I, Wurst R, Klinkenberg M, Scholz M, Euler T (2006) Yale: Rapid prototyping for complex data mining tasks. In: Proceedings of the 12th ACM SIGKDD international

conference on knowledge discovery and data mining (KDD-06) p 935–940 https://doi.org/10.1145/1150402.1150531

39 Ricker WE (1975) Computation and interpretation of biological statistics of fish populations. Bull Fish Res Board Can 191: 382 p.

40 Hilborn R, Walters CJ (1992) Quantitative fish stock assessment. Choice, dynamics and uncertainty. Chapman & Hall, New York, NY

41 Fix E, Hodges JL (1951) Discriminatory Analysis–Nonparametric discrimination consistency properties. Int Stat Rev 57: 238–247

42 Kohavi R (1995) A study of cross-validation and bootstrap for accuracy estimation and model selection. In: Mellish CS (ed) Proceedings of the 14th international joint conference on artificial intelligence, Montreal, Quebec. Morgan Kaufmann Publishers, San Mateo, CA, p 1137−1143

43 Breiman L (2001) Random forests. Mach Learn 45: 5−32

44 Polikar R (2006) Ensemble based systems in decision making. IEEE Circuits and Systems Magazine 6:21-45 Third Quarter 2006 doi:10.1109/MCAS.2006.1688199

45 Breiman L (1996) Bagging predictors. Mach Learn 24: 123−140

46 White ER, Schakner Z, Bellamy A, Srinivasan M (2022) Detecting population trends for US marine mammals. Conserv Sci Pract 4: e611 https://doi.org/10.1111/csp2.611

47 Northridge SP (1996) A review of marine mammal bycatch observer schemes with recommendations for best practice. JNCC report 219. Joint Nature Conservation Committee, Aberdeen, United Kingdom.

48 Vanhatalo J, Vetemaa M, Herrero A, Aho T, Tiilikainen R (2014) By-catch of grey seals (Halichoerus grypus) in Baltic fisheries—A Bayesian analysis of interview survey. PloS one 9:e113836

49 Bisack KD (1997) Harbor porpoise bycatch estimates in the US New England multispecies sink gillnet fishery: 1994–1995. Rep Int Whal Comm 47:705–714

50 Walsh WA, Kleiber, P, McCracken M (2002) Comparison of logbook reports of incidental blue shark catch rates by Hawaii-based longline vessels to fishery observer data by application of a generalized additive model. Fish Res 58:79–94 doi: 10.1016/S0165-7836(01)00361-7

51 Mangi SC, Smith S, Catchpole TL (2016) Assessing the capability and willingness of skippers towards fishing industry-led data collection. Ocean Coast Manag 134: 11–19

52 Emery TJ, Noriega R, Williams AJ, Larcombe J (2019) Changes in logbook reporting by commercial fishers following the implementation of electronic monitoring in Australian Commonwealth fisheries. Mar Policy 104: 135–145 doi: 10.1016/j.marpol.2019.01.018

53 Valtonen M, Palo JU, Aspi J, Ruokonen M, Kunnasranta M, Nyman T (2014) Causes and consequences of fine-scale population structure in a critically endangered freshwater seal. BMC Ecol 14:1–14

54 Sundell T, Kammonen JI, Mustanoja E, Biard V, Kunnasranta M, Niemi M, Nykänen M, Nyman T, Palo JU, Valtonen M, Paulin L, Jernvall J, Auvinen P (2023) Genomic evidence uncovers inbreeding and supports translocations in rescuing the genetic diversity of a landlocked seal population. Cons Gen 24: 155–165

55 Liukkonen L, Rautio A, Sipilä T, Niemi M, Auttila M, Koskela J, Kunnasranta M (2017) Long-term effects of land use on perinatal mortality in the Endangered Saimaa ringed seal population. Endang Species Res 34: 283–291

56 Taylor BL, Gerrodette T (1993) The uses of statistical power in conservation biology: The vaquita and northern spotted owl. Conserv Biol 7:489–500

57 Glemarec G, Königson S, Kindt-Larsen L (2021) Bycatch in Baltic Sea commercial fisheries: High-risk areas and evaluation of measures to reduce bycatch. HELCOM ACTION

58 Peckham SH, Diaz DM, Walli A, Ruiz G, Crowder LB, Nichols WJ (2007) Small-scale fisheries bycatch jeopardizes endangered Pacific loggerhead turtles. PloS one 2:e1041

59 Shester GG, Micheli F (2011) Conservation challenges for small-scale fisheries: Bycatch and habitat impacts of traps and gillnets. Biol Conserv 144:1673–1681

60 Lloret J, Cowx IG, Cabral HN, Castro M, Font T, Gonçalves JM, Gordoa A, Hoefnagel EW, Matić-Skoko S, Mikkelsen E, Morales-Nin B, Moutopoulos DK, Muñoz M, Santos MN, Pintassilgo P, Pita C, Stergiou KI, Ünal V, Veiga PA, Erzini K (2016) Small-scale coastal fisheries in European Seas are not what they were: Ecological, social and economic changes. Mar Policy 98:176–186

Figure_1 Click here to access/download;Figure;Figure 1.docx

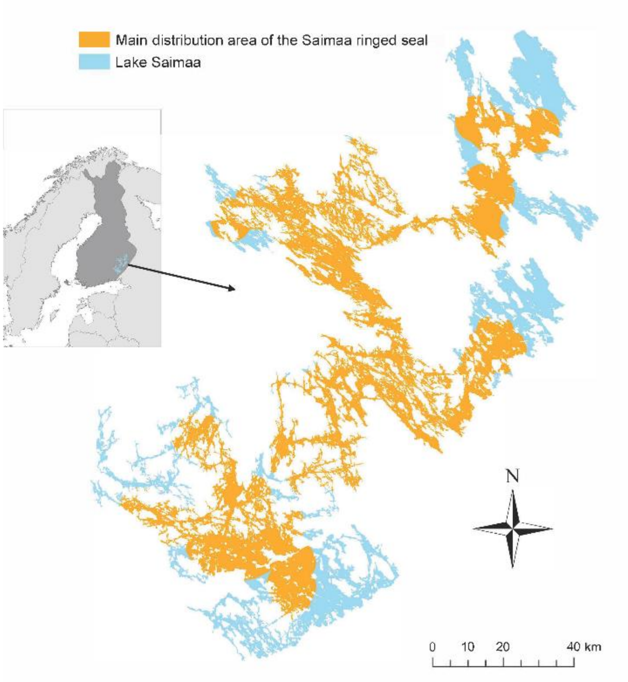

Figure 1. Lake Saimaa and the current main distribution area of the Saimaa ringed seal. Data from the Finnish National Land Survey 1:100 000 geographical map (downloaded 1/2015), MHPWF Saimaa ringed seal breeding lair sites in 2010–2022, and https://www.naturalearthdata.com/downloads/10m-cultural-vectors/10m-admin-0-countries/ (downloaded 2/2023).

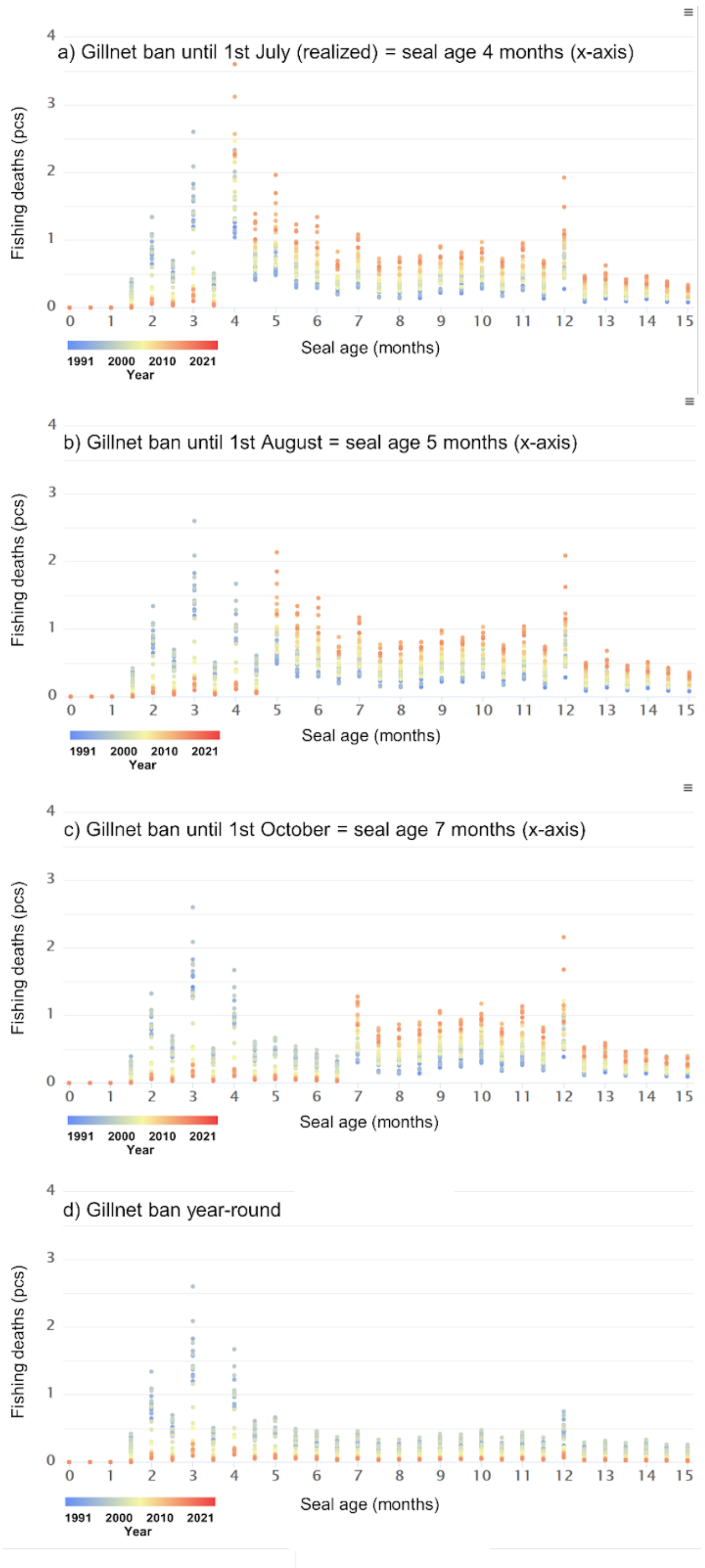

Figure 2. Estimated bycatch of Saimaa ringed seal juveniles (0 to 15 months) between 1991 and 2021: (a) the actual start of gillnet fishing, with the date 1st July corresponding to a ringed

seal age of four months, b) the start of gillnet fishing, with the date 1st August corresponding to a ringed seal age of five months, c) the start of gillnet fishing, with the date 1st October corresponding to a ringed seal age of seven months, and d) the year-round ban of gillnet fishing. For example, the first bycatch peak (y-axis) in the 1990s (blue colour) (Figure a) occurred in June due to the small coverage of closed areas (x-axis), after which the first bycatch peak (y-axis) moved forward to the beginning of July (x-axis), as the area closed to gillnet fishing increased from the beginning of the 2000s (yellowish–reddish colour),. In 1991, closed areas covered approximately 8% and, since the end of the 2010s, closed area coverage was approximately 95% of juvenile distribution areas.

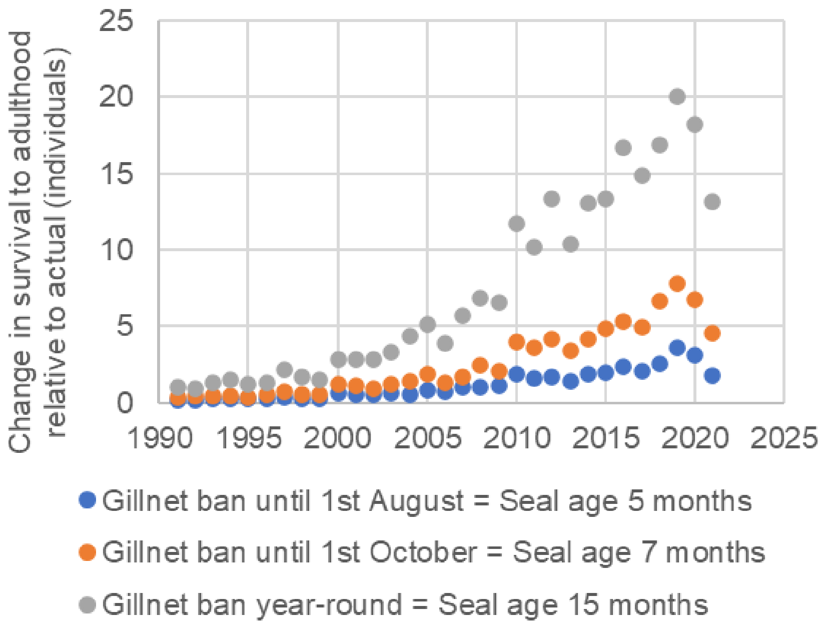

Figure 3. Change in juvenile survival to adulthood relative to actual survival (number of surviving individuals, y-axis) if temporal closures would have begun on 1st August (=1-month delay; age of ringed seal five months), 1st October (=3-month delay; age of ringed seal seven months), or if the temporal closure had been year-round from 1991 to 2021 (x-axis). For example, the interpretation of the blue dot on the right: moving the start of gillnet fishing forward by one month to the beginning of August (blue colour) in 2021 (x-axis) would have increased the survival of 0–15-month-olds by 1.79 individuals (y-axis). Increases in survival are instantaneous estimates, which means that the calculation does not account for the possible long-term cumulative effects of the change.

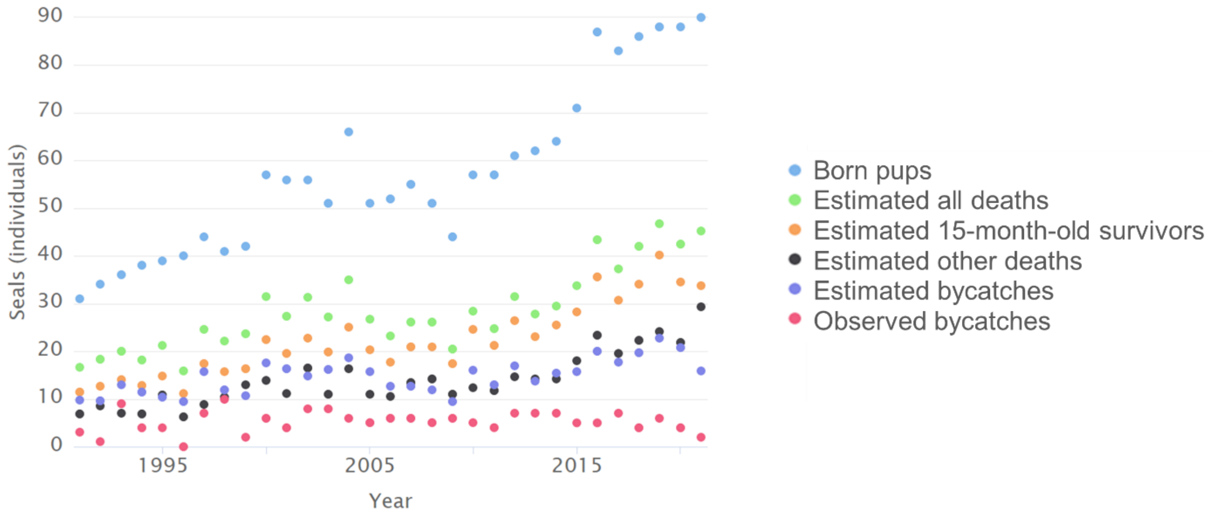

Figure 4. Number of born Saimaa ringed seal pups, estimated all deaths of max 15-month-old seals, estimated 15-month-old survivors, estimated other deaths, estimated bycatches, and observed bycatches (individuals) between 1991 and 2021.

Click here to access/download
Supporting Information
Supporting information_13thJune2024.docx
