# Supplementary material for: Effects of fishing restrictions on the recovery of the endangered Saimaa ringed seal (Pusa hispida saimensis) population
Source: PLoS One. 2024 Dec 5;19(12):e0311255. doi: 10.1371/journal.pone.0311255 (PMC11620628; doi:10.1371/journal.pone.0311255)
Supplement: Attachment — Submitted filename: Response to Reviewers.docx [file pone.0311255.s006.docx]

Dear Editor and reviewers,

Thank you for the very clear suggestions on how to improve the quality of the manuscript to meet the standards of PLOS ONE Journal. Please find below reviewer comments (left), and author responses (right) in two side-by-side tables. The line numbers in this ‘Response to Reviewers.docx’ file refer to the line numbers in the ‘Revised Manuscript with Track Changes.docx’ file.

| **REVIEWER comments from file: ‘PONE-D-24-24142_comments.pdf’** | **Authors corrections and comments** |
| --- | --- |
| Line 28: Replace ‘still existing’ with ‘continuing’ | Done |
| Line 49: Replace ‘is still’ with ‘remains’ | Done |
| Line 65: Replace hyphen ‘– ‘ with ‘and’ | Done |
| Line 67: Replace ‘greater’ with ‘primary’ | Done |
| Line 76: Add comma ‘,’ | Done |
| Line 89: Remove ‘al’ | Done |
| Line 99: Add ‘for marine mammals’ | Done |
| Line 100: Add ‘or more’ | Done |
| Lines 101 - 110: Add and remove sentences and citations as suggested | Done |
| Line 112: Replace ‘This study presents’ with ‘We present’ | Done |
| Line 120: Replace ‘(‘ with ‘of’ | Done |
| Line 122: Remove ‘of the Saimaa ringed seal’ | Done, and also the citation was transformed to number format ‘[37]’ |
| Line 127: Replace ‘labyrinthic’ with ‘labyrinthine’ | Done |
| Line 125: These don't seem to be presented in the paper. It would be helpful to me to have a sense of how well the model fits the (independent?) estimates. | Here the words ‘population size estimates’ referred to the lower sentence: ‘The starting assumption was that the 1990 population size of this study equalled that of MHPWF (mean 189, range 164–210; [26].’ To avoid further confusion, we removed these words ‘population size estimates’. |
| Line 134: Can you say how it was derived? | Done, added explanation and citation as follows: ‘(i.e. birth site locations % inside fishing restriction area, see also [13].’ |
| Line 142: Missing reference | Added reference |
| Lines 145-146: Replace ‘suffocations detected’ with ‘other carcasses determined’ | Done |
| Line 146: Add ‘to have drowned’ | Done |
| Line 150: Replace ‘calculated by subtracting from the’ with ‘the difference between’ | Done |
| Line 150: Add ‘and’ | Done |
| Table 1. This is a substantial number, and the largest single category. It seems important to note whether it could include any bycatch mortality. Is it mutually exclusive of 'Fishing' and 'Suffocation'?  If not, can you put any likely bounds on how much of it could be due to fishing? | Yes, in the Results section 3.3. we write: ‘The carcass data suggested an average 40% of the carcasses >0.5 months old that were found independent of fishing gear entanglements were judged to have died by suffocation (drowning) in 1991–2021.’  In other words, 40% is a carcass data-based multiplier (fishing mortality ‘guesstimate’) for over 0.5-month-old seals whose cause of death is ‘Unknown’. |
| Line 160: Replace ‘lair site diving surveys’ with ‘diving surveys of lair sites’ | Done |
| Line 174: Replace ‘pup’ with ‘seal’ | Done |
| Line 174: Replace ‘, first as pups’ with ‘as two age groups: ‘pups’ | Done |
| Line 175: Add comma ‘,’ | Done |
| Line 175: Replace ‘then as adults’ with ‘adults’ | Done |
| Line 177: Replace ‘individuals aged less than or equal to 15 months and older’ with ‘adults and pups’ | Done |
| Line 178: Replace ‘A’ with ‘This’ | Done |
| Line 178: Replace ‘emplyed here’ with ‘used’ | Done |
| Line 179: Replace ‘the’ with ‘The’ | We did not understand the reason for this change -> hence no replacement |
| Line 181: Replace ‘observed’ with ‘adult’ | Done |
| Line 182: Replace ‘over 15-month old’ with ‘adult’ | Done |
| Line 182: Replace ‘counted as one’ with ‘pooled in a’ | Done |
| Line 194: This is month and year of age, and not month and year of carcass collection, right? Maybe just say age to avoid confusion | Done, now referred to as ‘month and year of age’ |
| Lines 203-204: It seems clearer to refer to this as "age-constant" mortality rates"; it's not the distribution that's linear, it's the rate. | Done, now referred to as ‘age-constant mortality rates’ |
| Lines 215-223: Mention two alternative approaches and consideration of the pros and cons of the approach used here. | Done, three new sentences with main pros of two alternative approaches explained. |
| Lines 226-227: This seems like an arcane term that most readers will conflate with the concept of statistical confidence, rather than a distribution of proportions among categories | Done, we included ‘distribution of proportions among categories’ in parentheses, which probably helps readers to understand the results. |
| Line 231-232: If any of these might have died from fishery interactions, that should be acknowledged and, if possible, some bounds put on the potential bias induced by ignoring this. | Done, we now acknowledge this by extending the sentence as follows: ‘, of which some might have died from fishery interactions.’ |
| Lines 243-244: Unclear sentence | Done, we removed this sentence |
| Line 246: Replace ‘momentary’ with ‘the response in’ | Done |
| Line 248: Replace ‘reach until’ with ‘expire at’ | Done |
| Line 250-251: Replace ‘The word momentary here means that simulated change in survival does’ with ‘These responses in survival rate may be transitory because they do’ | Done |
| Lines 256-257: I don't think you can view the PBR approach in this way. When the PBR approach is applied to the management of a population, it is a means of ensuring a high probability that the population will trend toward or stay within the OSP range of abundance. It doesn't guarantee any particular growth rate. For example, the simulations used in the development of the PBR control rule allowed the time for recovery from 0.3K to MNPL to be up to 100 years (Wade 1998). | We replaced ‘are consistent with reaching the Saimaa ringed seal target growth rate’ with ‘ensure a high probability that the population will trend toward or stay within the OSP range of abundance’ |
| Line 263: Replace ‘as’ with ‘at’ | Done |
| Lines 264-266: There is nothing in the PBR approach about using survival rates, let alone a lower bound of a 60% CI for survival. The lower 60% CI bound concept in PBR is a means of deriving Nmin from the best estimate of N, when that is necessary. | We replaced ‘That is, for all adult cohorts in all years, we apply a lower limit of a 60% CI survival estimate.’ with ‘The lower 60% CI bound concept in PBR was used to derive Nmin from the best estimate of N.’ |
| Line 281: Replace ‘momentary’ with ‘,but possibly transitory, | Done |
| Lines 302-305: It may not be clear to many readers what "suffocation rate" means. I assume this means that 40% of the carcasses >0.5 months old that were found independent of fishing gear entanglements were judged to have died by suffocation. If so, just say something like that. | We replaced ‘40% suffocation rate for over 0.5-month-old seals found accidentally (mostly stranded seals)’ with ‘40% of the carcasses >0.5 months old that were found independent of fishing gear entanglements were determined to have died by suffocation’ |
| Line 304: Also, "drowning" might be a more apt word than "suffocation" | We added ‘drowning’ in parentheses |
| Line 303: Better word? It's not really an accident that they were found, correct? Is it that they were reported independently from mortalities reported by fishers? | Done, we replaced ‘accidentally’ with ‘found independent of fishing gear entanglements’ |
| Line 331: Replace ‘prominent’ with ‘frequent’ | Done |
| Line 331: It's not clear to me what this means or adds to the sentence. | Done, we removed words: ‘and causes additional mortality’ |
| Line 368: Replace ‘self-reported with ‘fisher-reported’ | Done |
| Line 369: Replace ‘suffocated’ with ‘fishery-independent drowned carcass recoveries’ | Done |
| Line 372-373: early in the manuscript you referred to these as "adults". It might make for smoother reading to use that same shorthand when describing and discussing the results. Similarly, "juveniles" might often be appropriate for referring to the other age class of your model | We rewrote this sentence to make it smoother reading |
| Line 361-362: can you say anything about whether delaying the fishing season would actually reduce total effort? | Not really, and this is probably one of the hardest topics to answer. We prefer leaving these less statistical guesstimates outside of this paper. |
| Line 378: Replace ‘good to observe’ with ‘noteworthy’ | Done |
| Lines 391-394: Replace sentence ‘Management actions would not be initiated at once due to an inability to detect a major decline in abundance.’ with ‘There are inherent delays in detection of a decline in abundance and management responses to a decline.’ | Done |
| Line 396: Replace ‘go’ with ‘become functionally’ | Done |
| Line 397: Remove ‘it is important to notice that’ | Done |
| Line 398: Remove ‘trend’ | Done |
| Lines 397-398: Add ‘Saimaa ringed seal’ | Done |
| Line 398: Add ‘annually’ | Done |
| Line 400: Remove ‘of the small Saimaa ringed seal population | Done |
| Line 399: Add ‘population’s | Done |

| **REVIEWER comments from file: ‘PONE-D-24-24142_reviewer copy.pdf’** | **Authors corrections and comments** |
| --- | --- |
| Line 40: Remove ‘s’ | Done |
| Lines 46-47: Remove ‘as a major driver in the decline of this endangered endemic seal [8], and bycatch has been identified’ | Done |
| Line 55: Remove ‘are suggested to’ | Done |
| Line 249: Remove comma ‘,’ | Done |
| Line 358: Remove ‘study showed that’ | Done |
| Lines 360-361: Remove ‘This is because’ | Done |
| Line 361: Remove ‘numbers’ | Done |
| Line 364-365: Remove ‘requested, and after 2016,’ | Done |
| Line 366: Remove ‘However, these’ | Done |
| Line 378: Remove ‘it is good to observe’ | In this sentence we followed the advice given by the other reviewer |
| Line 382: Remove ‘mortality’ | Done |
| Line 383: Remove comma ‘,’ | Done |
| Lines 391-394: MAYBE REWORD: While bycatch directly increases the risk of extinction, management responses likely would be delayed by our inability to detect a major population decline. | In this sentence we followed the advice given by the other reviewer |
| Line 403: Remove ‘studies have also indicated that’ | Done |
| Line 404: Remove ‘This is also the case’ | Done |
| Line 405: Remove ‘where’ | Done |
| Line 406: Remove ‘Additionally,’ | Done |
| Lines 406-407: Replace ‘with a’ with ‘accounting for’ | Done |
| Line 407: Remove ‘share in Lake Saimaa’ | Done |
| Figure 2: These panels are not easy to read. Perhaps, you want to omit this figure. Figure 3 nicely summarizes the effect of different closure dates. | We agree it’s a bit hard to read. On the other hand, it’s good to show the temporal between year and within season shifts in bycatch distribution. Hence, we would like to keep this figure (or e.g. transfer it to Appendix/Supplement if Editor insists that). Afterall, the other reviewer did not mention this and hence we assume it’s not that bad. |
| There were three question marks in the reviewer’s pdf version page 18.   | We assume that the reviewer’s Acrobat Reader/pdf-software has not been updated lately which may have caused a little poor printing quality of the text in page 18. The pdf-quality of the other reviewer was good. |
